# Midkine Increases Diagnostic Yield in AFP Negative and NASH-Related Hepatocellular Carcinoma

**DOI:** 10.1371/journal.pone.0155800

**Published:** 2016-05-24

**Authors:** Roslyn Vongsuvanh, David van der Poorten, Tristan Iseli, Simone I. Strasser, Geoffrey W. McCaughan, Jacob George

**Affiliations:** 1 Storr Liver Unit, Westmead Millennium Institute, University of Sydney at Westmead Hospital, Sydney, NSW, Australia; 2 AW Morrow Gastroenterology and Liver Centre, Royal Prince Alfred Hospital, Sydney, NSW, Australia; University of Navarra School of Medicine and Center for Applied Medical Research (CIMA), SPAIN

## Abstract

Robust biomarkers for population-level hepatocellular carcinoma (HCC) surveillance are lacking. We compared serum midkine (MDK), dickkopf-1 (DKK1), osteopontin (OPN) and AFP for HCC diagnosis in 86 HCC patients matched to 86 cirrhotics, 86 with chronic liver disease (CLD) and 86 healthy controls (HC). Based on the performance of each biomarker, we assessed a separate longitudinal cohort of 28 HCC patients, at and before cancer diagnosis. Serum levels of MDK and OPN were higher in HCC patients compared to cirrhosis, CLD and HC groups. DKK1 was not different between cases and controls. More than half of HCC patients had normal AFP. In this AFP-negative HCC cohort, 59.18% (n = 29/49) had elevated MDK, applying the optimal cut-off of 0.44 ng/ml. Using AFP ≥ 20 IU/ml or MDK ≥ 0.44 ng/ml, a significantly greater number (76.7%; n = 66/86) of HCC cases were detected. The area under the receiver operating curve for MDK was superior to AFP and OPN in NASH-HCC diagnosis. In the longitudinal cohort, MDK was elevated in 15/28 (54%) of HCC patients at diagnosis, of whom 67% had elevated MDK 6 months prior. **Conclusion:** AFP and MDK have a complementary role in HCC detection. MDK increases the diagnostic yield in AFP-negative HCC and has greater diagnostic performance than AFP, OPN and DKK-1 in the diagnosis of NASH-HCC. Additionally, MDK has a promising role in the pre-clinical diagnosis of HCC.

## Introduction

Hepatocellular carcinoma (HCC) represents a major global health challenge with rising incidence rates mirrored by a parallel increase in mortality [1, 2]. Whilst overall cancer death rates have decreased in the United States by 20% between 1990–2005, deaths from HCC have increased 27% [3, 4]. To combat this trend, surveillance guidelines for the early detection of HCC have been proposed [5, 6], but population level screening of at-risk individuals has not been realised for the majority. A challenge for effective population level HCC surveillance is the lack of a robust blood biomarker. Serum alpha-fetoprotein (AFP), the most widely used, has suboptimal performance with less than a fifth of early stage tumours presenting with elevated levels [7], and is therefore not currently recommended in American or European guidelines [5, 6]. An array of alternative markers have been proposed, however none have been sufficiently validated for routine practice [6].

One reason for the lack of a single specific and sensitive HCC biomarker is the highly heterogeneous nature of HCC at a molecular level [8] not only between individuals, but between tumours within the one individual, and even within a single tumour [1]. Hence, single biomarkers are unlikely to capture the complexity of pathways driving hepatocarcinogenesis. Consequently, a paradigm shift from searching for *the* single biomarker, to profiling a combination of biomarkers might be required to maximise diagnostic yield.

The proteins midkine (MDK), osteopontin (OPN) and dickkopf-1 (DKK1) have recently attracted attention for their purported superiority over AFP in the diagnosis of HCC. MDK is a heparin-binding growth factor, strongly expressed during embryogenesis whose expression is weak or undetectable in normal adult tissues [9]. In a gene expression profiling study, MDK was identified as one of 5 potential biomarkers for HCC [10]. Another study demonstrated that serum MDK is elevated in most HCC and may have a diagnostic role in AFP-negative and early stage tumours [11]. OPN, a phosphorylated glycoprotein has been associated with poor prognosis in HCC [12] and was found to be more sensitive than AFP for HCC diagnosis [13]. Finally, DKK1, an antagonist of the Wnt signalling pathway [14], was demonstrated to complement AFP in HCC diagnosis particularly in AFP-negative cancers [15].

A direct comparison of MDK, OPN and DKK1 with AFP has not previously been undertaken. Furthermore, a major shortcoming in the literature is the paucity of longitudinal studies to determine the value of these biomarkers in detecting early stage, asymptomatic cancers, a prerequisite for effective surveillance. Finally, the specificity of these markers when compared to non-HCC liver diseases and healthy controls has not been adequately ascertained. We assessed the performance of serum MDK, OPN, DKK1 and AFP alone or in combination for the diagnosis of HCC, as compared to their serum levels in patients with cirrhosis, chronic liver disease or healthy controls. We further evaluated the capacity of these markers to detect early stage HCC in a cohort of at-risk patients followed prospectively.

## Methods

### Part I- Cross-sectional study

#### Study design and patient characteristics

This case-control study involved four independent groups comprising a total of 344 participants recruited from a single tertiary liver clinic in Sydney, Australia. The HCC group comprised 86 patients with tumours diagnosed by characteristic radiological appearances on 4-phase CT or MRI according to the European Association for the Study of the liver (EASL) guidelines [6], or by histology. Clinical staging of HCC was according to the Barcelona Clinic Liver Cancer (BCLC) system. Serum was taken at the time of diagnosis, prior to the initiation of treatment.

The HCC cases were age and sex matched (+/- 10 years) to three additional cohorts comprising patients with cirrhosis, chronic liver disease without cirrhosis, and healthy controls. The cirrhosis group comprised 86 individuals with cirrhosis of any aetiology. Cirrhosis was diagnosed by histopathology where possible, or on the basis of clinical, laboratory and/or imaging evidence, including transient elastography, thrombocytopenia <150x10^9^/L and/or imaging findings of a macronodular liver, splenomegaly or reduced portal vein blood flow. The chronic liver disease (CLD) group included 86 patients with chronic hepatitis B (HBV) in the absence of cirrhosis. Patients in the cirrhosis and chronic liver disease groups were undergoing 6 monthly HCC surveillance with no evidence of HCC at the time blood was collected for the study and for a minimum follow-up of 6 months thereafter. The healthy control (HC) group comprised 86 individuals recruited through advertisements in local newspapers and at the hospital. All had normal physical examinations and liver tests, negative viral hepatitis serology and no history of liver disease.

The study protocol was approved by the Human Ethics Committee of the Sydney West Area Health Service. Written informed consent was obtained from all participants.

#### Measurement of biomarkers

In all subjects, serum was collected in a plastic ethylene diamine tetra acetic acid tube (EDTA), centrifuged and stored at -80°C until testing. Serum MDK was measured by ELISA using a commercial kit supplied by Cellmid, Sydney AU. Serum AFP was measured using a chemiluminsecent microparticle immunoassay (Abbott Diagnostics, Illinois US). DKK-1 and OPN levels were determined using a multiplex analyte detection assay (Milliplex MAP). All assays were performed according to the manufacturers’ instructions and values reported in ng/ml for MDK (limit of detection, LOD, 0.008; coefficient of variation, CV = 4.5%), OPN (LOD 0.0377; CV = 2%) and DKK-1 (LOD 0.0014; CV = 7%) and in IU/ml for AFP (LOD, 1.66; CV = 7.5%).

#### Statistical analysis

Statistical analysis was carried out using SPSS version 21.0 (SPSS Inc., Chicago, IL). Descriptive statistics were reported as median, mean, standard deviation and range as appropriate. Kruskal-Wallis non-parametric analysis of variance (ANOVA) was used to determine statistically significant differences in the four biomarkers across the four groups. Pairwise comparison with Bonferroni correction was used to compare differences between groups. The strength of association between continuous variables was reported using Spearman rank correlations. Receiver operating characteristics (ROC) curves were used to evaluate the sensitivity, specificity and area under the curve (AUC) with 95% confidence interval (CI). Optimal diagnostic threshold values were determined by calculating the minimum distance to the top of the ROC curve. The analyses were repeated after stratifying for aetiology and early-stage HCC (BCLC 0-A). To investigate whether the combined use of the biomarkers was better than one single marker, a new variable which predicted probability for HCC was generated using binary logistic regression. This function was then subjected to ROC analysis and its performance evaluated against AFP.

### Part II- Longitudinal Study

If a biomarker was found to be of potential diagnostic utility based on the results of the cross-sectional study, we further investigated whether it was elevated in samples prior to HCC diagnosis. For this, in a separate longitudinal cohort, 28 patients with prospectively collected blood samples who subsequently developed HCC were assessed. Blood samples were collected at the time of HCC diagnosis and were available from 6 months prior to diagnosis. HCC was excluded at 6 months prior to diagnosis based on negative imaging by ultrasound. These 28 cases were age and sex matched (+/- 10 years) to three independent control groups, also with longitudinal blood samples collected at time 0 and 6 months prior. The control groups comprised 28 patients with HBV cirrhosis, 28 patients with HCV cirrhosis and 28 patients with chronic HBV without cirrhosis. All controls had no evidence of HCC during the study period and within a minimum period of 6 months thereafter. Cases and controls were recruited from two tertiary hospitals in Sydney, Australia.

## Results

### Part I- Cross-sectional Study

#### Patient characteristics

The clinical characteristics of the HCC cases and controls are summarized in Table 1. The groups were well matched for age and gender, except that the HCC cohort was older than the respective controls.

**Table 1:** Clinical characteristics of HCC patients and controls.

		HCC (n = 86)	Cirrhotics (n = 86)	*P* value (HCC vs cirrhotics)	Chronic liver disease (n = 86)	*P* value (HCC vs CLD)	Healthy control (n = 86)	*P* value (HCC vs HC)
**Demographics**	Male (n, %)	75 (87)	75 (87)	1.0	75 (87)	1.0	75 (87)	1.0
	Age (years)	62.2 (11.4)	58.8 (9.9)	**0.041**	58.42 (8.5)	0.015	54.2 (9.2)	<0.001
**Ethnicity (n,%)**	Caucasian	55 (64)	49 (57)	0.349	9 (10.5)	<0.001	71 (82.6)	**0.006**
	East Asian	13 (15.1)	12 (14)	0.829	56 (65.1)	<0.001	9 (10.5)	0.361
	Middle Eastern	10 (11.6)	20 (23.3)	**0.044**	10 (11.6)	1.0	2 (2.3)	**0.017**
	South Asian	3 (3.5)	3 (3.5)	1.0	7 (8.1)	0.192	4 (4.7)	0.700
	Polynesian	2 (2.3)	1 (1.2)	0.56	2 (2.3)	1.0	0	0.155
	African	3 (3.5)	1 (1.2)	0.31	2 (2.3)	0.650	0	0.081
**Etiology (n, %)**	HBV	14 (16.3)	23 (26.7)	0.095	86 (100)	<0.001	0	-
	HCV	42 (47.7)	43 (50)	0.76	0	-	0	-
	Alcohol	11 (12.8)	7 (8.1)	0.319	0	-	0	-
	NASH	16 (18.6)	10 (11.6)	0.202	0	-	0	-
	OTHER	4 (4.7)	3 (3.5)	0.700	0	-	0	-
**Cirrhosis (n, %)**	Presence of cirrhosis	76 (88.4)	86 (100)	**0.001**	0	-	0	-
	Child-Pugh A	55 (64)	78 (90.7)	**<0.001**	0	-	0	-
	Child-Pugh B	14 (16.3)	6 (7.0)	0.057	0	-	0	-
	Child-Pugh C	7 (8.1)	2 (2.3)	0.087	0	-	0	-
**Metabolic risk factors**	Diabetes (n, %)	35 (40.7)	29 (33.7)	0.344	10 (11.6)	**<0.001**	0	-
	BMI	28.6	29.4	0.349	25.4	**<0.001**	26.8	<0.001

Results are expressed as mean (standard deviation) or frequency (percentage).

Abbreviations: HCC, hepatocellular carcinoma; CLD, chronic liver disease; HC, healthy controls; HBV, hepatitis B virus; HCV, hepatitis C virus; NASH, non-alcoholic steatohepatitis; BMI, body mass index

*P* values were calculated using the independent variable t test or Pearson chi-square test.

#### Serum biomarker levels in the HCC and control groups

**Fig 1:**
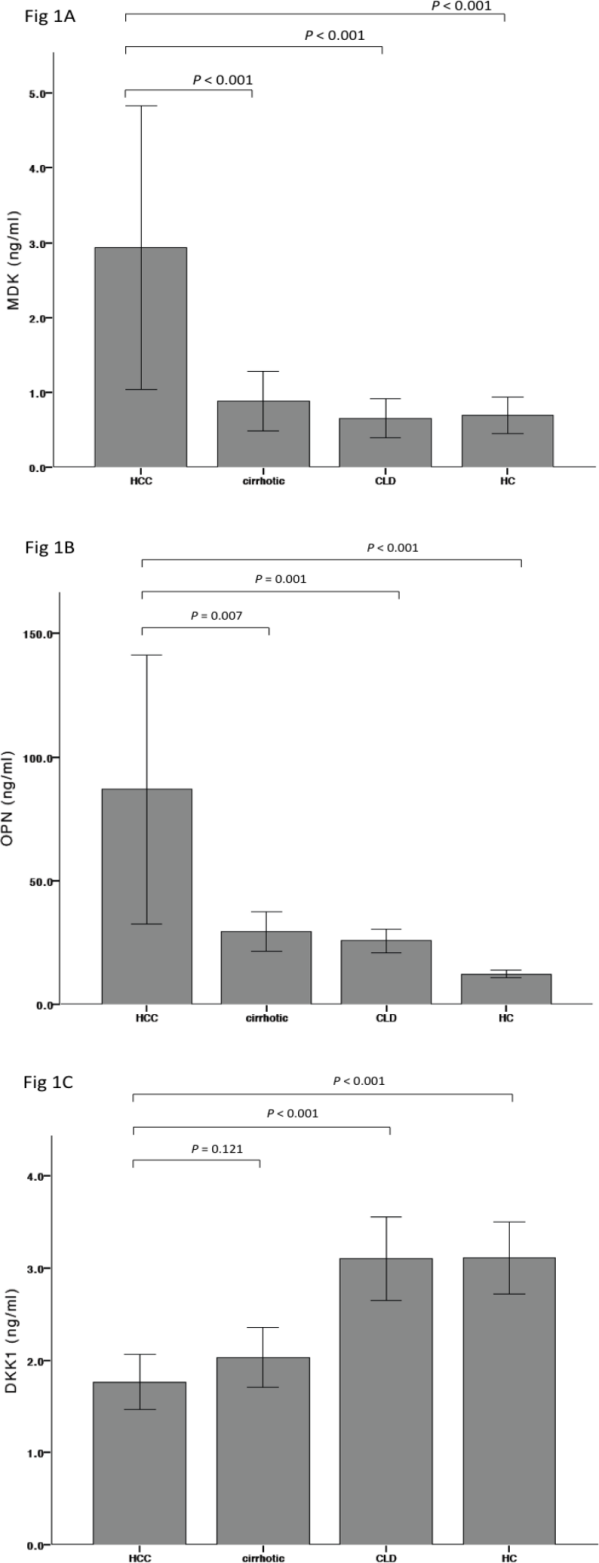
Mean serum MDK, OPN and DKK1 concentrations in HCC and control groups. A–C) MDK, OPN and DKK1 levels respectively for HCC and control groups. Mean values and 95% confidence intervals are shown. Abbreviations: MDK, midkine; OPN, osteopontin; DKK1, dickopff-1; HCC, hepatocellular carcinoma; CLD, chronic liver disease; HC, healthy controls. Comparison between HCC and the control groups was performed by independent variable t tests after log transformation of non-parametric data.

Serum levels of MDK, OPN, DKK1 and AFP in the HCC and control groups are displayed in Fig 1. MDK was significantly higher in HCC (mean 2.93 ng/ml, SD 0.96 ng/ml; median 0.57 ng/ml, IQR 0.4–1.4) than in cirrhosis (mean 0.88 ng/ml, SD 0.20 ng/ml; median 0.39 ng/ml, IQR 0.3–0.7), CLD (mean 0.65 ng/ml, SD 0.13 ng/ml; median 0.35 ng/ml, IQR 0.2–0.6) and HC (mean 0.70 ng/ml, SD 0.12; median 0.40 ng/ml, IQR 0.3–0.5) (*P* < 0.0001 for all). Even after stratifying for liver disease severity (based on Child-Pugh status), mean values of MDK remained significantly higher in the HCC group compared to the cirrhosis group (S1 Table). OPN was also elevated in HCC (mean 86.98 ng/ml, SD 27.37 ng/ml; median 35.7ng/ml, IQR 17.4–51.6) compared to cirrhosis (29.47 ng/ml, SD 4.0 ng/ml; median 23.08 ng/ml, IQR 16.5–31.6), CLD (mean 25.72 ng/ml, SD 2.46 ng/ml; median 22.78 ng/ml, IQR 14.5–31.0;) and HC (mean 12.30 ng/ml, SD 0.77 ng/ml; median 11.7 ng/ml, IQR 6.9–1.6) (*P* < 0.01 for all). There was no significant difference in DKK1 levels between HCC cases (mean 1.76 ng/ml, SD 0.15 ng/ml; median 1.4 ng/ml, IQR 0.9–2.1) and cirrhotics (mean 2.031 ng/ml, SD 0.163 ng/ml; median 1.68 ng/ml, IQR 1.0–2.6), CLD (mean 3.10 ng/ml, SD 0.23 ng/ml; median 2.52 ng/ml, IQR 1.9–3.8) or HC (mean 3.11 ng/ml, SD 0.20 ng/ml; median 2.83 ng/ml, IQR 1.9–3.6) (*P* = 0.44), suggesting that DKK1 has limited value as a biomarker for HCC diagnosis. AFP was significantly higher in HCC (mean 2913.0 ng/ml, SD 1424.7 ng/ml; median 15.3 IU/ml, IQR 4.0–12.6) than cirrhosis (mean 6.48 ng/ml, SD 1.32 ng/ml; median 3 IU/ml, IQR 1–2.6) and CLD (mean 2.54 ng/ml, SD 0.10; median 2.0 IU/ml; IQR 2.0–3.0). Using a cut-off of 20 IU/ml, more than half (56.98%; n = 49/86) of HCC patients were AFP-negative.

The association between the biomarkers and various clinicopathological features of HCC were analysed (Table 2). MDK, OPN and AFP levels were not associated with HCC aetiology. MDK was associated with the greatest number of aggressive clinical and tumour characteristics such as poor Child-Pugh status (*P* = 0.01), advanced BCLC stage (*P* = 0.006), vascular invasion (*P* = 0.007) and high tumour number (*P* = 0.007). The mean value of MDK in patients with metastases was lower (1.01 ng/ml) compared to patients without metastases (3.08 ng/ml). This result, however, is not statistically significant (*P* = 0.998) and interpretation is limited by the small number of patients in our HCC cohort who had metastases (n = 6/86). AFP was associated with vascular invasion (*P* <0.0001) and tumour number (*P* = 0.03). OPN was also associated with high tumour number (*P* = 0.02). There was a trend towards higher MDK, OPN and AFP in patients who had a time to progression of less than 12 months, although this was not statistically significant.

**Table 2:** Mean MDK, OPN, DKK1 and AFP levels in HCC patients according to various clinical parameters.

		n	OPN	MDK	DKK1	AFP
Age	<55	29	54.75	1.067	2.443	493.74
	>55	57	103.38	3.882	1.418	2128.17
	*P* value		0.272	0.218	**< 0.001**	0.665
Sex	Male	76	94.28	3.91	1.696	1959
	Female	10	31.50	11.31	2.281	10160.34
	*P* value		0.533	**0.04**	0.312	0.404
Aetiology	HBV	14	80.36	1.17	3.03	8182.67
	HCV	41	52.68	2.39	1.32	383.94
	NASH	16	131.19	5.59	1.89	63355.49
	ETOH	11	183.43	3.45	1.73	1683.91
	Other	4	19.67	2.62	1.45	2.93
	*P* value		0.836	0.834	**0.01**	0.227
Child Pugh	A	55	46.27	1.36	1.59	2673.76
	B-C	21	110.28	8.11	1.84	4858.19
	*P* value		0.582	**< 0.001**	0.790	0.721
BCLC	0-A	36	67.388	0.799	1.783	299.889
	B-D	50	101.087	4.469	1.750	4794.510
	*P* value		0.718	**0.006**	0.996	0.035
Tumour size	< = 5cm	62	41.32	2.91	1.55	758.97
	>5cm	24	204.94	3.00	2.31	8477.72
	*P* value		**0.009**	0.218	**0.034**	0.382
Tumour number	<5	73	78.8	2.548	1.2144	838.030
	> = 5	13	103.06	3.688	1.946	6991.510
	*P* value		**0.018**	**0.007**	0.345	**0.032**
Vascular invasion	Yes	13	167.95	6.20	2.22	14852.73
	No	72	72.68	2.38	1.70	797.04
	*P* value		0.167	**0.007**	0.458	**< 0.001**
TTP	<12m.o	52	90.32	4.12	1.693	2296.02
	>12 m.o	17	37.45	1.22	1.819	1405.907
	*P* value		0.849	0.402	0.480	0.229
Metastases	Yes	6	294.30	1.01	2.39	4481.85
	No	80	71.43	3.08	1.72	2795.38
	*P* value		0.192	0.998	0.208	0.117

Abbreviations: MDK, midkine; OPN, osteopontin; DKK1, dickopff-1; AFP, alpha-fetoprotein; BCLC, Barcelona Clinic Liver Cancer Staging Classification; TTP, time to progression.

*P* values using analysis of variance (ANOVA) or independent t test after log transformation of non-parametric data.

#### Performance of the novel biomarkers compared to AFP

**Fig 2:**
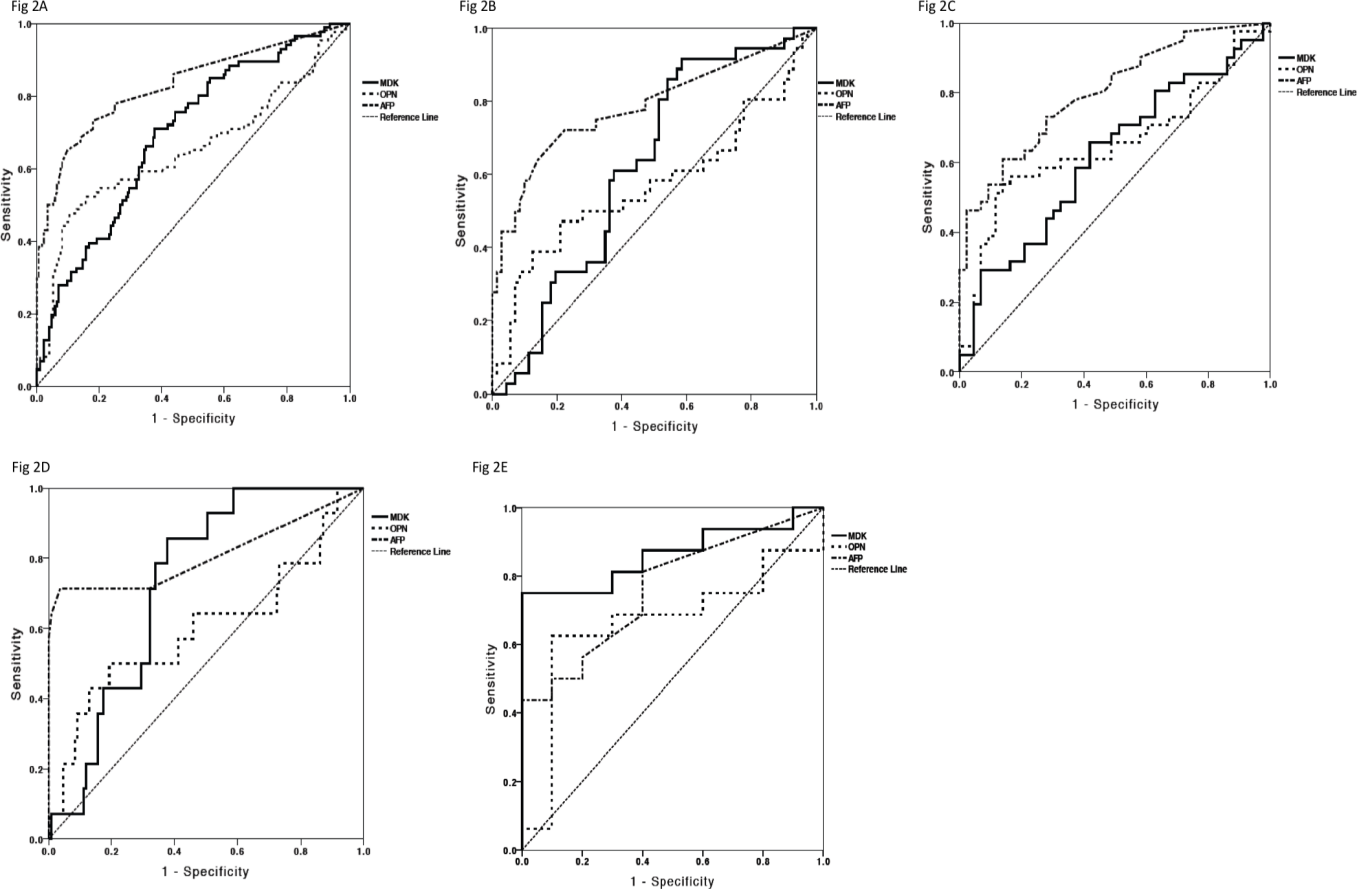
Comparison of the diagnostic performances of serum MDK, OPN and AFP. **A) All HCC patients versus non-HCC patients (cirrhotics and chronic liver disease). B) Early HCC patients (BCLC 0-A) versus non-HCC patients (cirrhotic and chronic liver disease). C) HCV-HCC patients versus HCV-cirrhotics. D) HBV-HCC patients versus HBV-cirrhotics and chronic HBV patients. E) NASH-HCC patients versus NASH cirrhotic patients.** A) ROC curve for MDK, OPN and AFP for all patients with HCC (n = 86) versus patients with cirrhosis (n = 86) or chronic liver disease (n = 86). B) ROC curve for MDK, OPN and AFP for all patients with early stage (BCLC 0-A) HCC (n = 36) versus patients with cirrhosis (n = 36) or chronic liver disease (n = 36). C) ROC curve for MDK, OPN and AFP for all patients with HCV-related HCC (n = 42) versus all patients with HCV-related cirrhosis (n = 43). D) ROC curve for MDK, OPN and AFP for all patients with HBV-related HCC (n = 14) versus all patients with HBV-related cirrhosis (n = 23) or chronic hepatitis B (n = 86). E) ROC curve for MDK, OPN and AFP for all patients with NASH-related HCC (n = 16) versus all patients with NASH-related cirrhosis (n = 10). Abbreviations: ROC, receiver operating characteristics; MDK, midkine; OPN, osteopontin; DKK1, dickopff-1; AFP, alpha-fetoprotein; HCC, hepatocellular carcinoma; CLD, chronic liver disease; HBV, hepatitis B virus; HCV, hepatitis C virus; NASH, non-alcoholic steatohepatitis.

We next evaluated the performance of the four biomarkers compared to AFP in discriminating HCC from non-HCC. DKK1 was omitted from these analyses given that there was no significant difference in DKK1 levels between HCC and controls as shown above. AFP (> 20 IU/ml) had a greater area under the ROC curve (AUROC 0.83; 95% CI 0.77–0.89) than MDK (0.70; 95% CI 0.63–0.76) and OPN (0.65; 95% CI 0.57–0.73) (Fig 2A), suggesting that AFP is superior to these biomarkers for HCC diagnosis. At a cut-off of 20 IU/ml, the sensitivity of AFP was 43.0%, specificity 96.5%, positive predictive value (PPV) 86.0% and negative predictive value (NPV) 77.2%. These results are comparable to the diagnostic performance of AFP quoted in the literature with a sensitivity of 39–65% and specificity 76–94% [16]. The optimal diagnostic cut-off for MDK based on the ROC curve was 0.44 ng/ml, with a sensitivity of 70.9% and specificity 62.2% (PPV 48.0% and NPV 80.9%). The optimal cut-off for OPN was 33.36 ng/ml giving a sensitivity of 54.7% and specificity 79.7% (PPV 37.0% and NPV 72.9%).

We next explored the diagnostic efficacy of the biomarkers in various HCC subgroups using AUROC. In detecting early stage (BCLC 0-A) HCC, AFP remained superior (0.79; 95% CI 0.69–0.89) to OPN (0.57; 95% CI 0.45–0.70) and MDK (0.63; 95% CI 0.52–0.73) (Fig 2B). In distinguishing HCV or HBV-associated HCC from cirrhosis (Fig 2C and 2D), AFP continued to have superior diagnostic performance compared to the novel biomarkers. Interestingly, when NASH-related HCC was compared to NASH cirrhosis, MDK had a greater AUROC (0.86; 95% CI 0.72–1.0) compared to AFP (0.76; 95% CI 0.58–0.95) and OPN (0.66; 95% CI 0.44–0.88), suggesting that MDK may have a role in the diagnosis of NASH-related HCC (Fig 2E). The sensitivities, specificities and predictive values of the biomarkers in the HCC subgroup analyses are available in S2 Table.

The optimal diagnostic cut-off for MDK based on the ROC curve was 0.44 ng/ml (sensitivity 70.9%, specificity 62.2%) and for OPN was 33.36 ng/ml (sensitivity 54.7%, specificity 79.7%). To determine whether a combination of biomarkers could improve performance for HCC diagnosis, AFP, MDK and OPN were entered into a binary logistic regression model from which only MDK and AFP were found to be significantly associated with HCC diagnosis and from which the following equation was derived: 3*logAFP + logMDK. When this combined score was compared to AFP in HCC diagnosis, the AUROC was only marginally improved compared to AFP alone (0.846 vs 0.831), suggesting that combining biomarkers did not significantly improve the diagnosis of HCC compared to either test alone.

The role of serum MDK in AFP-negative (<20 IU/ml) HCCs was also investigated. In patients with HCC, 56.98% (n = 49/86) had normal AFP. Of these 49 patients with AFP-negative HCC, 59.18% (n = 29/49) had elevated MDK using the optimal diagnostic cut-off of 0.44 ng/ml. Using a criteria of AFP ≥ 20 IU/ml or MDK ≥ 0.44ng/ml, a significantly greater number (76.7%; n = 66/86) of HCC cases were detected, supporting a complementary role of MDK to AFP in HCC diagnosis. Spearman’s rank correlation was performed to determine the relationship between serum MDK and AFP, resulting in a rho value of 0.257 (*P* = 0.017). This suggests that MDK and AFP reflect different, independent pathways in HCC development.

### Part II- Longitudinal Study

#### Serum MDK in the pre-clinical diagnosis of HCC

We have shown in the cross-sectional study that serum MDK could have a diagnostic role in AFP-negative HCC and may differentiate NASH-HCC from non-malignant liver disease. In a pilot study, we therefore further investigated whether serum MDK levels were raised in samples prior to HCC diagnosis. Of 28 HCC patients evaluated longitudinally, MDK was elevated in 15/28 (54%) of patients at diagnosis, of whom 67% had elevated MDK 6 months prior (Table 3). AFP was elevated (≥ 20 IU/ml) in 14/28 (50%) of patients at diagnosis, of whom 50% had elevated levels 6 months prior. Of the remaining 50% (n = 14) with AFP-negative HCC, 6/12 (50%) had elevated MDK at diagnosis and 4/12 (33%) had elevated MDK 6 months prior. This suggests that MDK may have a role in the pre-clinical diagnosis of HCC, however further studies with larger numbers are needed to validate this finding.

**Table 3:** Frequency of elevated serum MDK and AFP at HCC diagnosis and 6 months prior to diagnosis.

Biomarker	Frequency of elevated biomarker at HCC diagnosis, n (%)	Frequency of elevated biomarker at 6 months pre-HCC diagnosis in patients with elevated levels at diagnosis, n (%)
MDK ≥ 0.44 ng/ml	15/28 (54)	10/15 (67)
AFP ≥ 20 IU/ml	14/28 (50)	7/14 (50)

Abbreviations: MDK, midkine; AFP, alpha-fetoprotein; HCC, hepatocellular carcinoma.

## Discussion

There is an unmet clinical need for improved serum HCC biomarkers to allow for objective and reproducible assessments in surveillance programs and for early diagnosis [17]. To date, HCC biomarkers have been disappointing with a lack of validated diagnostic performance and a paucity of longitudinal studies in at-risk cohorts. In this large case-control study involving cross-sectional and longitudinal components, we compared three novel biomarkers- OPN, MDK and DKK1- to AFP. We found that serum MDK may have an important complementary role to AFP, as it increases the diagnostic yield in AFP-negative HCC and the presence of either elevated AFP or MDK increases the sensitivity of HCC detection. Serum MDK was also superior to AFP in the diagnosis of NASH-HCC and was associated with more aggressive tumour clinicopathological features. Importantly, we show that MDK is elevated in pre-clinical tumour samples and therefore may have a role in the early detection of HCC.

A significant limitation to the use of AFP for HCC surveillance is the rate of AFP-negative HCC. Up to 50% of small HCCs do not secrete AFP and even with larger lesions, 20% are not associated with elevated levels [18]. Consistently in our cohort, more than half (57%) of HCCs were negative for AFP. In this group however, 60% had elevations in MDK using an optimal cut-off of 0.44 ng/ml. Applying a criteria of either AFP ≥ 20 IU/ml *or* MDK ≥ 0.44ng/ml, a significantly greater number (77%) of tumours would have been detected compared to 43% if AFP was used alone. This finding is even more noteworthy as the majority (72%) of these cases were early stage (BCLC 0-A) tumours- a group difficult to detect with current surveillance strategies but important to target for potentially curative treatment. These results confirm those of Zhu et al [11] who found that the sensitivity of MDK in AFP-negative HCC could reach as high as 89.2%. The higher sensitivity in the Zhu et al study probably reflects the fact that they had a lower percentage of early stage tumours (49%) and were from a single centre in China with mainly HBV-related HCC.

A second novel finding from this study is that serum MDK has superior diagnostic performance to AFP in the detection of NASH-HCC (AUROC 0.86; 95% CI 0.72–1.0 versus 0.76; 95% CI 0.58–0.95). Although our NASH-HCC cohort is small and requires validation, this observation has important clinical implications. With burgeoning rates of obesity and diabetes, NASH will become an increasingly important cause of HCC worldwide [19]. Surveillance criteria for HCC in NASH is not well defined and is complicated by the fact that one third of NASH-HCCs occur in non-cirrhotic livers [20]. The availability of a serum biomarker with greater diagnostic accuracy for NAFLD-HCC than AFP may influence the way in which high-risk NAFLD/NASH patients are screened as they comprise a significant proportion (between 30–40%) of the adult population in affluent nations [21, 22]. In the NAFLD cohort, population level ultrasound screening is clearly impractical. That MDK would be a superior marker for NASH-HCC than AFP has biological plausibility. MDK is induced by inflammation [23] with potent pro-inflammatory activities including chemotaxis of neutrophils and macrophages [24]. Importantly, MDK is expressed in adipocytes and levels are increased in the adipose tissues of obese mice and in the serum of obese humans [25]. It is plausible therefore that early in NAFLD-HCC pathogenesis, MDK has increased expression in tumour tissue or that MDK expression in adipocytes contributes directly to tumour development. Our hypothesis, paves way for further studies to investigate mechanisms linking MDK with NAFLD related liver tumors.

A limitation in biomarker research is the relative paucity of longitudinal studies examining the capacity of a biomarker to detect pre-clinical cancers. In the cross-sectional component, we established a potential role for MDK in HCC detection. As a next step, we performed a pilot study to investigate whether MDK was elevated in pre-HCC diagnosis using serum samples in an at-risk cohort followed prospectively. This represents a phase III study design as defined by the National Cancer Institute Guidelines for the development of biomarkers for detection of cancer [26]. 50% of HCC patients had elevated AFP (≥20 IU/ml) at diagnosis and within this AFP-positive cohort, 50% had elevated levels 6 months prior despite negative ultrasound imaging. Our results are consistent with the study of Lok et al [27] in which of 39 HCC patients, 57% had high AFP 6 months prior to diagnosis. We likewise observed that MDK was elevated in approximately half of patients at diagnosis, however compared to AFP, a higher proportion (67%) had elevated MDK 6 months prior. These results suggest that MDK may have a role in the detection of pre-clinical HCC and could be used as a tool to guide follow-up of high-risk patients. For example, for high-risk patients on a screening program, elevations in MDK may necessitate a shorter interval of imaging follow-up than the 6 months currently recommended.

Based on the present data, neither serum OPN or DKK1 can be recommended as routine biomarkers for HCC surveillance or screening. Serum OPN, while elevated in HCC had inferior performance to AFP and MDK with an AUROC of 0.66. Furthermore, by binary logistic regression model, whilst AFP and MDK were associated with HCC diagnosis, OPN was not. Serum DKK1 likewise had inferior performance to all other biomarkers tested and did not differentiate HCC cases from controls. This result is unexpected in light of the study by Shen et al [15] suggesting that DKK1 could distinguish HCC from non-malignant liver disease and complement AFP in HCC diagnosis. Despite their cohort size (n = 831), the latter retrospective study is limited by an exclusively Chinese cohort with more than 80% of cases due to HBV. Thus, as we demonstrate, their results may not be replicated in other ethnicities or translate to other HCC aetiologies. This is not altogether surprising with previous experience with DCP, PIVKA and AFP-L3 showing promise in Asian populations but not replicated in other countries [28].

Our study has several strengths. First, it examines the biomarkers in parallel, which has not been previously reported. The cohort of well-matched cases and controls with HCC of different aetiologies enabled us to perform detailed sub-analyses. Although the numbers in individual HCC subgroups were small, our population was sufficiently heterogeneous to perform subgroup analyses which has not been possible in some larger but more homogenous study populations [13, 15, 29]. Importantly, whilst few studies in the literature include prospective follow-up of high-risk patients with serial biomarker determinations [30], we had access to longitudinal serum samples from those who subsequently did and did not develop HCC. Although this cohort is small in number, it is the largest to examine MDK prospectively in pre-diagnostic samples. One study by Hung et al [31] attempted longitudinal measurements of MDK in at-risk patients, however only two patients in their cohort developed de novo HCC. There was no rise in MDK prior to diagnosis in these two patients. Furthermore, other longitudinal biomarker studies to date such as by Sterling et al [30] and Shang et al [13], have had comparable sample sizes to ours (n = 46 and n = 22 respectively).

In conclusion, AFP and MDK may have a complementary role in HCC surveillance and screening. MDK increases the diagnostic yield in AFP-negative HCC and the presence of either elevated AFP or MDK increases the sensitivity of HCC detection. In practice, if the AFP is negative (<20 IU/ml) but MDK elevated (≥0.44 ng/ml), a higher index of suspicion for an AFP-negative tumour is warranted. If initial ultrasound imaging is negative, these patients might require a shorter surveillance interval or more diagnostic modalities such as CT or MRI. MDK is also superior to AFP in the diagnosis of NASH-related HCC and this finding postulates an exciting novel role for MDK in NASH-related carcinogenesis that warrants further investigation.

## Supporting Information

S1 Table Biomarker levels stratified according to Child-Pugh status. Abbreviations: MDK, midkine; OPN, osteopontin; DKK1, dickopff-1; AFP, alpha-fetoprotein; HCC, hepatocellular carcinoma; Childs, Child-Pugh. *P values using independent t-test after log transformation of non-normal data.
(DOCX)

S2 Table Diagnostic performance of MDK, OPN and AFP in HCC subgroups. Abbreviations. AFP, alpha-fetoprotein; MDK, midkine; OPN, osteopontin; HCC, hepatocellular carcinoma; HBV, hepatitis B virus; HCV, hepatitis C virus; NASH, non-alcoholic steatohepatitis.
(DOCX)

## Abbreviations

HCC: hepatocellular carcinoma
AFP: alpha-fetoprotein
MDK: midkine
OPN: osteopontin
DKK1: dickkopf-1
EASL: European Association for the Study of the liver
BCLC: Barcelona Clinic Liver Cancer
CLD: chronic liver disease
HBV: chronic hepatitis B
HC: healthy control
EDTA: ethylene diamine tetra acetic acid tube
LOD: limit of detection
CV: coefficient of variation
ANOVA: analysis of variance
ROC: receiver operating characteristics
AUC: area under the curve
CI: confidence interval
NASH: non-alcoholic steatohepatitis
NAFLD: non-alcoholic fatty liver
DCP: des-gamma-carboxy prothrombin
