# Supplementary material for: Midkine Increases Diagnostic Yield in AFP Negative and NASH-Related Hepatocellular Carcinoma
Source: PLoS One. 2016 May 24;11(5):e0155800. doi: 10.1371/journal.pone.0155800 (PMC4878793; doi:10.1371/journal.pone.0155800)
Supplement: S1 Table — Abbreviations: MDK, midkine; OPN, osteopontin; DKK1, dickopff-1; AFP, alpha-fetoprotein; HCC, hepatocellular carcinoma; Childs, Child-Pugh. *P values using independent t-test after log transformation of non-normal data. (DOCX) [file pone.0155800.s001.docx]

**S1 table. Biomarker levels stratified according to Child-Pugh status**

|  |  | HCC | Cirrhosis | *P* value* |
| --- | --- | --- | --- | --- |
| MDK ng/ml | Childs A | 1.36 | 0.89 | **0.04** |
|  | Childs B-C | 8.11 | 0.76 | **0.03** |
| OPN ng/ml | Childs A | 46.27 | 29.99 | 0.05 |
|  | Childs B-C | 110.28 | 24.45 | 0.38 |
| AFP IU/ml | Childs A | 2673.76 | 5.3 | **<0.001** |
|  | Childs B-C | 4958.19 | 17.63 | 0.14 |
| DKK1 IU/ml | Childs A | 1.59 | 2.09 | 0.03 |
|  | Childs B-C | 1.84 | 1.48 | 0.83 |

Abbreviations: MDK, midkine; OPN, osteopontin; DKK1, dickopff-1; AFP, alpha-fetoprotein; HCC, hepatocellular carcinoma; Childs, Child-Pugh.

**P* values using independent t-test after log transformation of non-normal data
