# Supplementary material for: Midkine Increases Diagnostic Yield in AFP Negative and NASH-Related Hepatocellular Carcinoma
Source: PLoS One. 2016 May 24;11(5):e0155800. doi: 10.1371/journal.pone.0155800 (PMC4878793; doi:10.1371/journal.pone.0155800)
Supplement: S2 Table — Abbreviations. AFP, alpha-fetoprotein; MDK, midkine; OPN, osteopontin; HCC, hepatocellular carcinoma; HBV, hepatitis B virus; HCV, hepatitis C virus; NASH, non-alcoholic steatohepatitis. (DOCX) [file pone.0155800.s002.docx]

**S2 table. Diagnostic performance of MDK, OPN and AFP in HCC subgroups**

|  | | AUC (95% CI) | Sensitivity (%) | Specificity (%) | PPV (%) | NPV (%) |
| --- | --- | --- | --- | --- | --- | --- |
| Early stage HCC | |  |  |  |  |  |
|  | AFP | 0.79 (0.69-0.89) | 33.3 | 97.2 | 85.7 | 74.5 |
|  | MDK | 0.63 (0.52-0.73) | 61.1 | 62.5 | 44.0 | 75.9 |
|  | OPN | 0.57 (0.45-0.70) | 47.2 | 76.4 | 48.6 | 74.0 |
| HBV-HCC | |  |  |  |  |  |
|  | AFP | 0.81 (0.64-0.98) | 42.9 | 1.0 | 100.0 | 93.2 |
|  | MDK | 0.73 (0.63-0.83) | 78.6 | 62.4 | 22.2 | 97.1 |
|  | OPN | 0.60 (0.42-0.79) | 42.9 | 80.7 | 25.0 | 92.6 |
| HCV-HCC | |  |  |  |  |  |
|  | AFP | 0.80 (0.71-0.89) | 53.7 | 86.0 | 78.6 | 66.1 |
|  | MDK | 0.62 (0.50-0.74) | 63.4 | 58.1 | 60.0 | 64.1 |
|  | OPN | 0.65 (0.53-0.77) | 56.1 | 81.4 | 74.2 | 66.0 |
| NASH-HCC | |  |  |  |  |  |
|  | AFP | 0.76 (0.58-0.95) | 30.0 | 100.0 | 100.0 | 45.5 |
|  | MDK | 0.86 (0.72-1.0) | 68.8 | 100.0 | 100.0 | 66.7 |
|  | OPN | 0.66 (0.44-0.88) | 62.5 | 80.0 | 83.3 | 57.1 |

Abbreviations: AFP, alpha-fetoprotein; MDK, midkine; OPN, osteopontin; HCC, hepatocellular carcinoma; HBV, hepatitis B virus; HCV, hepatitis C virus; NASH, non-alcoholic steatohepatitis.
